# Over-Expression of a Melon Y3SK2-Type *LEA* Gene Confers Drought and Salt Tolerance in Transgenic Tobacco Plants

**DOI:** 10.3390/plants9121749

**Published:** 2020-12-10

**Authors:** Samuel Aduse Poku, Peter Nkachukwu Chukwurah, Htut Htet Aung, Ikuo Nakamura

**Affiliations:** Laboratory of Plant Cell Technology, Chiba University, 648 Matsudo, Chiba 271-8510, Japan; adusepokusamuel5@gmail.com (S.A.P.); petlinks2000@gmail.com (P.N.C.); htetaunghtut@gmail.com (H.H.A.)

**Keywords:** abiotic stress, late embryogenesis abundant protein, stress tolerance

## Abstract

Climate change, with its attendant negative effects, is expected to hamper agricultural production in the coming years. To counteract these negative effects, breeding of environmentally resilient plants via conventional means and genetic engineering is necessary. Stress defense genes are valuable tools by which this can be achieved. Here we report the successful cloning and functional characterization of a melon Y3SK2-type dehydrin gene, designated as *CmLEA-S*. We generated *CmLEA-S* overexpressing transgenic tobacco lines and performed in vitro and in vivo drought and salt stress analyses. Seeds of transgenic tobacco plants grown on 10% polyethylene glycol (PEG) showed significantly higher germination rates relative to wild-type seeds. In the same way, transgenic seeds grown on 150 mM sodium chloride (NaCl) recorded significantly higher germination percentages compared with wild-type plants. The fresh weights and root lengths of young transgenic plants subjected to drought stress were significantly higher than that of wild-type plants. Similarly, the fresh weights and root lengths of transgenic seedlings subjected to salt stress treatments were also significantly higher than wild-type plants. Moreover, transgenic plants subjected to drought and salt stresses in vivo showed fewer signs of wilting and chlorosis, respectively. Biochemical assays revealed that transgenic plants accumulated more proline and less malondialdehyde (MDA) compared with wild-type plants under both drought and salt stress conditions. Finally, the enzymatic activities of ascorbate peroxidase (APX) and catalase (CAT) were enhanced in drought- and salt-stressed transgenic lines. These results suggest that the *CmLEA-S* gene could be used as a potential candidate gene for crop improvement.

## 1. Introduction

Over the years, accumulated evidence has shown that climate change has taken its toll on agriculture and has made the task of ending hunger, achieving food security, improving nutrition, and promoting sustainable agriculture even more difficult [1]. Climate variability and extremes, possibly the result of climate change, are the key drivers behind the recent rise in global hunger and are one of the major causes of the severe food crises. This variability has resulted in severe droughts and soil salinity and threatens to erode most of the gains made towards achieving global food security [2].

The breeding of environmentally resilient plants is one major way by which a sustainable agricultural production system can be achieved. Molecular breeding involves the introduction of genes encoding stress responsive proteins such as the late embryogenesis abundant (LEA) proteins, osmoprotectant synthetizing enzymes, heat shock proteins (HSP) and transcription regulators such as *NAC*, *WRKY* and *MYB* genes that can confer resistance to diverse environmental stresses into plants by means of genetic engineering.

The late embryogenesis abundant (LEA) proteins are a large family of hydrophilic proteins that accumulate in developing seeds to ensure protection against water deficit stress. Research has revealed LEA genes to be present in most plants and in different plant tissues. LEA proteins are characterized by different conserved sequence motifs and are highly rich in alanine, glycine and serine residues [3]. The proteins play vital roles in normal plant growth and in abiotic stress responses. The expression of LEA proteins was discovered to be upregulated in response to environmental stress factors such as water deficit, salinity, and cold stress. The proteins have been implicated in several roles, including the protection of cellular structures from desiccation induced damage [4], the sequestration of ions [5], and the folding of denatured proteins [6]. Moreover, they can also act as chaperone proteins to protect cells against membrane damage [7]. The group 2 members of the LEA family of proteins, also known as dehydrins (DHNs) are highly hydrophilic proteins. These proteins can be classified into five subgroups; YnSKn, Kn, SKn, YnKn, and KnS depending on the number of conserved Y, S and K motifs [8]. Dehydrins can act as molecular chaperones in plants under stress. Plants exposed to stresses accumulate reactive oxygen species (ROS) such as hydrogen peroxide and super oxides, which may induce oxidative damage and cell death [9]. As molecular chaperones, dehydrin proteins may perform the dual function of providing cellular protection and performing damage repairs in cells exposed to stresses, thereby providing stability to biomolecules and proteins [10]. These functions may help to protect plants from the harmful effects of oxidative damage.

Quite a number of studies have been carried out on *LEA* genes in the Cucurbitaceae family. One study investigated *LEA* genes in *Cucumis sativus* and discovered that a dehydrin gene denoted as *CsLEA54* might have influenced the adaptation of cucumber to drought stress [11]. A study of another cucumber *LEA* gene, *CsLEA11*, in *E. coli* cells revealed that the overexpression of the gene enhanced cell viability and conferred tolerance to heat and cold stresses [12]. Genome wide comparative studies of *Citrullus lanatus* (watermelon) and *Cucumis melo* (melon) revealed a total of 73 *LEA* genes for watermelon and 61 *LEA* genes for melon. In the same study, gene expression analysis of selected *LEA* genes in root and leaf tissues of drought-stressed watermelon and melon plants revealed that *ClLEA-12-17-46* and *CmLEA-42-43* genes were upregulated after drought application [13].

*Cucumis melo*, an important member of the Cucurbitaceae family, has the significant potential of becoming a model plant for the study of vital traits in fruit development, given its morphological, physiological, and biochemical diversity in flavor development and textural changes during fruit ripening [14]. Given the relatively hardy nature of melons [15,16,17] we hypothesized that the plant may contain potential useful candidate dehydrin genes that could be used in the molecular breeding of plants. To the best of our knowledge, there has been very little focus on the identification and characterization of useful candidate dehydrin genes in the melon plant. Here we report the functional characterization of a drought and salt stress responsive dehydrin gene denoted as *CmLEA-S.* The *CmLEA-S* gene conferred drought and salt stress tolerance to transgenic tobacco plants by enhancing osmoprotectant accumulation, limiting lipid peroxidation and improving the antioxidation machinery of transgenic tobacco plants.

## 2. Results

### 2.1. Sequence Alignment and Phylogenetic Analysis of CmLEA-S

**Figure 1:**
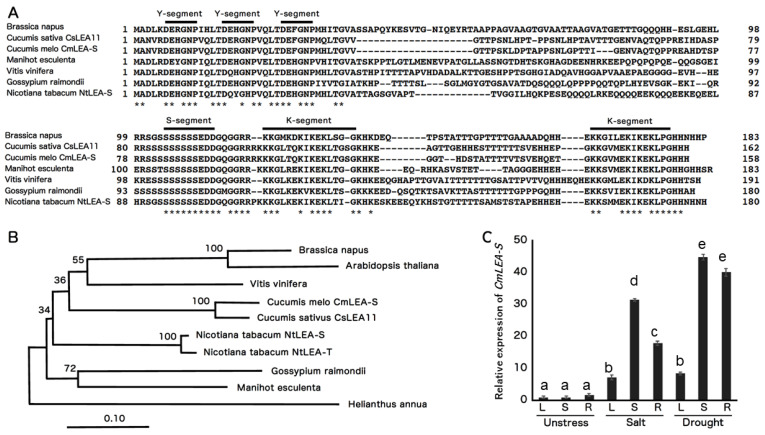
Bioinformatics and expression analysis of melon CmLEA-S. (**A**) Sequence alignment of CmLEA-S protein with other plant Y3SK2-type LEA proteins. Bold lines indicate conserved amino acid sequences of Y-, S-, and K-segment motifs. Asterisks (*) indicate identical amino acids. (**B**) Phylogenetic relationships among plant Y3SK2-type LEA proteins using neighbor-joining method. *Cucumis melo CmLEA-S*, *Cucumis sativus CsLEA11* and Y3SK2-type LEA proteins of *Brassica napus*, *Arabidopsis thaliana*, *Manihot esculenta*, *Gossypium raimondii*, *Vitis vinifera*, *Nicotiana tabacum* and *Helianthus annua*. (**C**) Expression analysis of *CmLEA-S* in leaf (L), shoot (S) and root (R) tissues of melon plants under salt (300 mM NaCl) and drought stress condition. Fold change in expression was computed relative to the rate of *CmLEA-S* expression in unstressed melon leaves. Data are means of triplicates from three independent experiments. Error bars indicate ± SD. Different letters indicate significant difference (*p* < 0.05) according to Duncan’s test.

*CmLEA-S* clone (570 bp) was amplified from the cDNA of melon leaves via RT-PCR. The *CmLEA-S* gene was predicted to encode a protein of 158 amino acid long with a molecular weight of 16.9 kDa. The deduced CmLEA-S protein contained three Y-segments, one S-segment and two K-segments (Y3SK2, Figure 1A). A phylogenetic tree generated with the amino acid sequences of CmLEA-S and other Y3SK2-type dehydrins from different plants showed the CmLEA-S protein shared a closer relationship with *Vitis vinifera* LEA protein but was distantly related with the LEA protein of *Helianthus* species (Figure 1B).

### 2.2. Expression Analysis of CmLEA-S in Melon

The expression level of the *CmLEA-S* gene was upregulated in the leaf, shoot and root tissues of salt- and drought-stressed melon plants relative to unstressed plants (Figure 1C). Transcript accumulation in plants under drought stress was higher in all tissues tested compared to salt-stressed and unstressed plants (Figure 1C). On the average, drought-stressed tissues accumulated 12 times more *CmLEA-S* transcripts than salt-stressed tissues and 30 times more transcripts than unstressed tissues. The highest levels of *CmLEA-S* expression were observed in drought-stressed shoots and roots of melon plants and the lowest expression levels in unstressed shoots and leaves (Figure 1C). *CmLEA-S* expression increased by 43-fold in drought-stressed shoots and 39-fold in drought stressed-roots relative to the expression levels in unstressed tobacco leaves which was used as a standard. These results suggest that the *CmLEA-S* gene is an abiotic stress responsive gene.

### 2.3. Transformation and Southern Blot Analysis of Tobacco Plants

**Figure 2:**
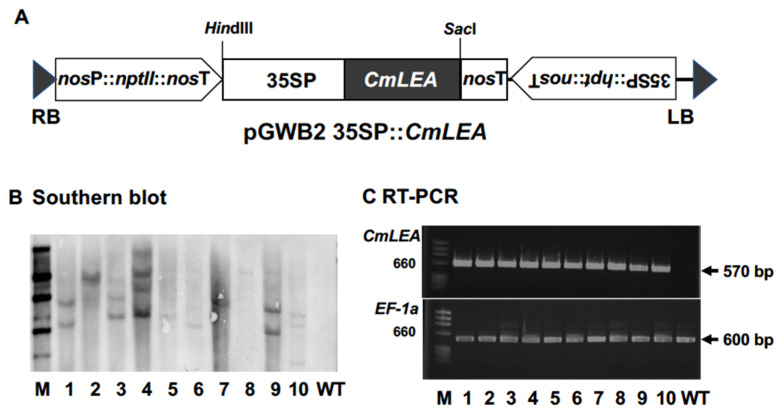
Transformation and molecular analysis of tobacco lines. (**A**) Schematic representation of the T-DNA region of pGWB2: *CmLEA-S* binary vector. *CmLEA-S* gene was under the control of the CaMV 35S promoter and the nopaline synthetase (*nos*) terminator between kanamycin and hygromycin selective marker cassettes. RB: right border, LB: left border. (**B**) Southern blot analysis of *hpt* transgene in transgenic tobacco lines. M: marker, WT: wild type. (**C**): RT-PCR analysis of *CmLEA-S* (570 bp) and *EF-1 a* (560 bp as internal control) transcripts in transgenic tobacco lines.

The *CmLEA-S gene* was inserted under the control of CaMV 35S promoter, between kanamycin and hygromycin selectable marker cassettes, in the pGWB2 plasmid to produce the pGWB2 35SP: *CmLEA-S* binary vector (Figure 2A). *A. tumefaciens* bearing pGWB2 35SP: *CmLEA-S* was inoculated to tobacco leaf discs. Regenerated transgenic tobacco plants were subjected to Southern blot analysis and RT-PCR. The Southern blot results reveal that four transgenic plants had a single copy of the integrated gene (Figure 2B). The RT-PCR results, on the other hand, confirm the expression of the *CmLEA-S* gene (570 bp) and *EF-1a* (560 bp, internal control) in transgenic tobacco lines (Figure 2C). Lines 2, 5 and 6 were selected for further analysis.

### 2.4. Stress Tolerance Analysis at Germination Stage

**Figure 3:**
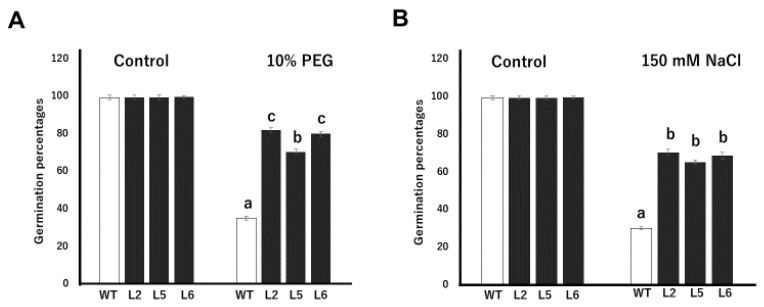
Tolerance assays against drought and salt stresses in transgenic tobacco lines at the germination stage. (**A**) Seed germination rate of wild-type and transgenic tobacco lines on filter paper soaked with 10% PEG. (**B**) Seed germination rate of tobacco lines on MS supplemented with 150 mM NaCl. WT; wild-type L2; line 2 L5; line 5 L6; line 6 Data are means of triplicates from three independent experiments. Error bars indicate ± SD. Bars with different letters represent significant difference (*p* < 0.05) according to Duncan’s test.

The germination profiles of wild-type and transgenic plants under normal and drought conditions were studied by growing seeds on filter papers soaked in water and 10% PEG, respectively. There were no significant differences between the germination capacities of wild-type and transgenic lines under normal conditions. However, under drought stress, the germination rates of transgenic lines were significantly higher than the wild-type line. On the average, transgenic lines showed about a 121% increase in germination rate, relative to the wild-type (Figure 3A). Transgenic line 2 showed the highest germination rate, recording a mean germination percentage of 82%. Transgenic lines 5 and 6, on the other hand, recorded mean germination percentages of 70.3% and 80%, respectively. The wild-type line recorded the lowest germination rate under drought conditions, with a mean germination rate of 35%. The germination rates of wild-type and transgenic seeds on normal MS medium and MS medium containing 150 mM NaCl were also compared. We observed no significant differences in the rate of seed germination on normal MS medium (Figure 3B). Under saline conditions however, the germination rates of transgenic seeds were significantly higher than wild-type seeds. Transgenic lines 2, 5 and 6 recorded mean germination rates of 70.3%, 65.2% and 68.7% respectively. The wild-type line, on the other hand, recorded the lowest germination percentage, with a germination average of 30%.

### 2.5. In Vitro-Stress Tolerance Assay at Seedling Stage

**Figure 4:**
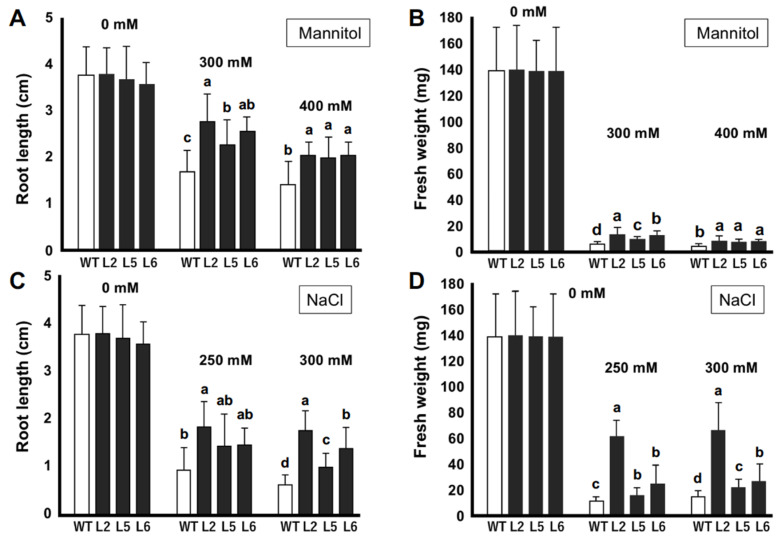
In vitro stress tolerance assays of transgenic tobacco seedlings. (**A**) Root lengths (cm) of tobacco plants under normal and mannitol-induced osmotic stress conditions. (**B**) Fresh weights (mg) of tobacco plants under normal and mannitol-induced osmotic stress conditions. (**C**) Root lengths (cm) of tobacco plants under normal and salt-stress conditions. (**D**) Fresh weight (mg) of tobacco plants under normal and salt-stress conditions. WT; wild-type L2; line 2 L5; line 5 L6; line 6. Data are means of triplicates from three independent experiments. Error bars indicate ± SD. Different letters indicate significant difference (*p* < 0.05) according to Duncan’s test.

To study the effects of *CmLEA-S* overexpression on the stress tolerance capacities of tobacco plants under osmotic conditions, 2 week-old T1 tobacco plants were grown on normal MS media and MS media supplemented with 300/400 mM mannitol. After 2 weeks, transgenic lines 2, 5 and 6 showed 22.1%, 28.9% and 26.0% reduction in root lengths (compared with the root lengths of individual lines grown on normal MS medium), respectively, when grown with 300 mM mannitol (Figure 4A). The wild-type line on the other hand showed as high as 54% reduction in root length under the same condition. A similar trend was observed for the root lengths of plants grown with 400 mM mannitol. The fresh weights of tobacco lines were also drastically affected by 300 and 400 mM mannitol (Figure 4B). However, all three transgenic lines showed significantly higher mean fresh weights compared with the wild-type line.

We analyzed the growth of tobacco seedlings under normal and saline conditions (Figure 4C,D). No significant differences were observed in the growth of tobacco plants under normal conditions. However, it was observed that transgenic lines 2, 5 and 6 showed 52%, 59% and 58% reductions in root length, respectively, with 250 mM NaCl compared with a 73% reduction in root length for the wild-type line (Figure 4C). We observed similar patterns in the growth of plant roots grown with 300 mM NaCl. The fresh weights of transgenic lines were also significantly higher than those of the wild-type line under saline conditions (Figure 4D). Transgenic line 2 showed the highest mean fresh weight with 250 and 300 mM NaCl. The wild-type line, on the other hand, showed the least average mean fresh weight under both conditions.

### 2.6. Drought Tolerance Assay on Soil

**Figure 5:**
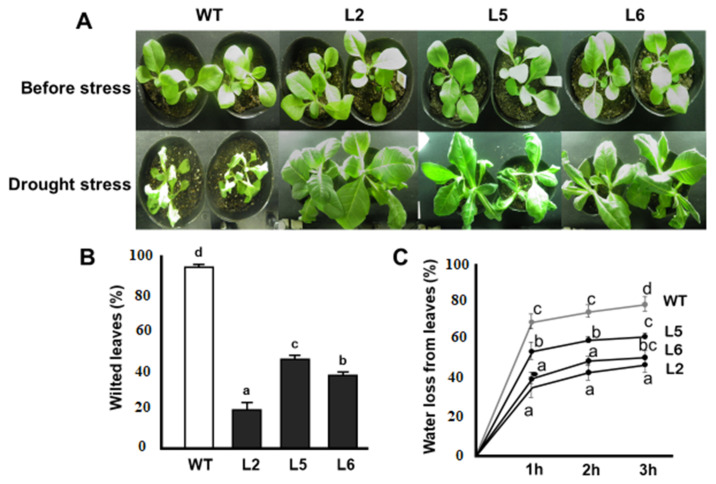
Responses of *CmLEA-S* transgenic tobacco lines to drought stress. (**A**) Phenotypes of tobacco lines before and after drought stress treatment. (**B**) Percentage of wilted leaves of tobacco lines after drought stress. (**C**) Rate of water loss in detached leaves of tobacco lines during dehydration. WT; wild-type L2; line 2 L5; line 5 L6; line 6. Data are means of triplicates from three independent experiments. Error bars indicate ± SD. Different letters show significant difference (*p* < 0.05) according to Duncan’s test.

The stress tolerance capacities of transgenic plants overexpressing the *CmLEA-S* gene was assayed under greenhouse conditions. Under drought stress, the growth of wild-type plants was highly repressed (Figure 5A). Furthermore, the majority of the leaves of wild-type plants showed severe signs of wilting (Figure 5B). Transgenic lines, on the other hand, showed better growth profiles and had comparatively fewer wilted leaves (Figure 5A,B). The rate of water loss in detached leaves of plants over a time span of 3 h was also investigated. It was observed that the extent of water loss in the wild-type line was significantly higher at each point than that of transgenic lines (Figure 5C). At the end of the experiment, the wild-type line had lost approximately 80% of its initial water content (Figure 5C). Transgenic lines, on the other hand, showed relatively lower rates of water loss, with lines 2, 5 and 6 losing approximately 35%, 60% and 40% of their initial water contents, respectively (Figure 5C).

### 2.7. Salt Stress Assay on Soil

**Figure 6:**
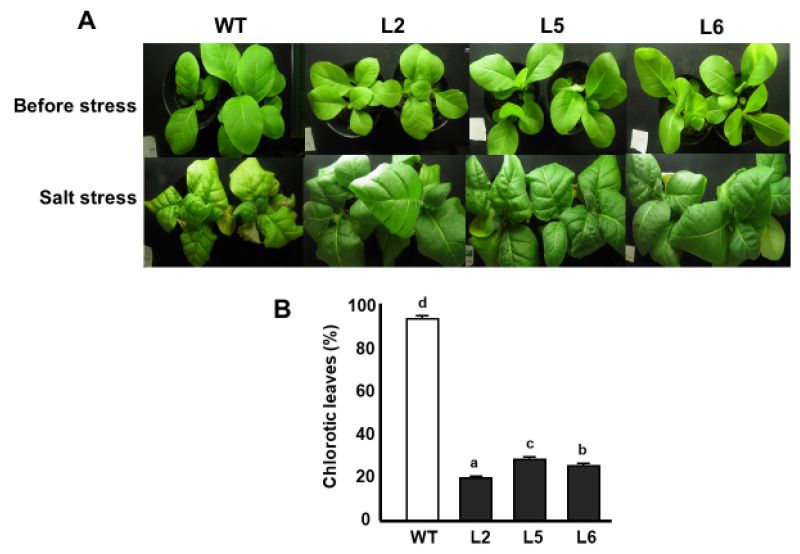
Responses of tobacco lines to salt stress. (**A**) Phenotypes of tobacco lines subjected to salt stress. (**B**) Percentage of leaves showing signs of chlorosis after salt stress treatment. WT; wild-type L2; line 2 L5; line 5 L6; line 6. Data are means of triplicates from three independent experiments. Error bars indicate ± SD. Different letters represent significant difference (*p* < 0.05) according to Duncan’s test.

Salt stress assays were performed on 5 week-old tobacco plants. The results of salt stress assay in vivo show that wild-type plants experienced stunting in growth compared with transgenic lines. Furthermore, the majority of the leaves of wild-type plants were found to be chlorotic, whereas relatively fewer leaves of transgenic plants displayed signs of chlorosis. Over 80% of the leaves of wild-type plants became chlorotic. In the case of transgenic lines, Line 5 showed the highest level of chlorosis of about 50% followed by Line 6 with a chlorosis rate of 40%. Line 2 showed the lowest level of chlorosis, with a 20% rate of chlorosis (Figure 6A,B).

### 2.8. Measurements of Malondialdehyde (MDA) and Proline Contents

**Figure 7:**
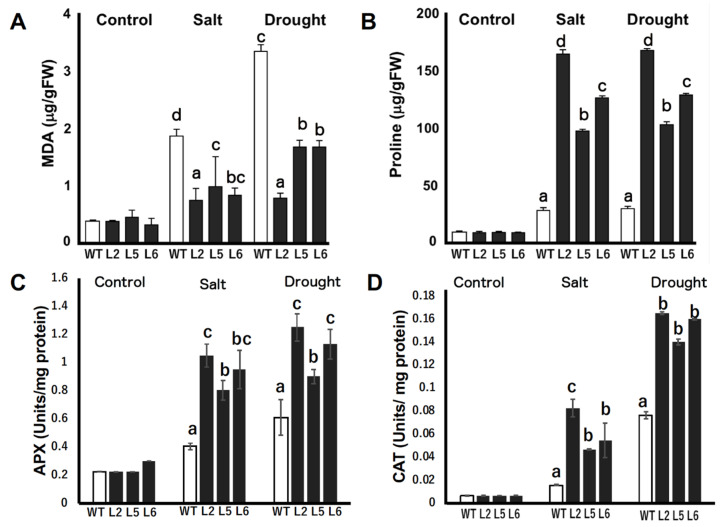
Relative concentration of malondialdehyde (MDA), proline, and enzymatic activities of ascorbate peroxidase (APX) and catalase (CAT) in wild-type and *CmLEA*-*S* overexpressing tobacco plants under normal and stressed conditions (**A**) MDA contents of unstressed and stressed tobacco plants. (**B**) Proline contents of unstressed and stressed tobacco plants. (**C**) Enzymatic activity of APX in tobacco plants under normal and stressed conditions. (**D**) Enzymatic activity of CAT in tobacco plants under normal and stressed conditions. WT; wild-type L2; line 2 L5; line 5 L6; line 6. Data are means of triplicates from three independent experiments. Error bars indicate ± SD. Different letters in graphs indicate significant differences between treatments (*p* < 0.05) according to Duncan’s test.

The relative contents of MDA and proline were determined and compared in the leaves of wild-type and transgenic lines under stressed and non-stressed conditions. Our results reveal that there were no significant differences in the MDA contents of tobacco plants grown under normal conditions (Figure 7A). However, the MDA contents of wild-type plants grown under drought and saline conditions were significantly higher than that of transgenic lines. On average, the MDA content of wild-type plants under stressed conditions was about twice that of transgenic lines under the same conditions.

The relative proline contents of the leaves of tobacco plants grown under normal conditions were not significantly different (Figure 7B). However, under drought and saline conditions, transgenic plants accumulated significantly higher amounts of proline compared with wild-type plants. Transgenic line 2 accumulated the highest amount of proline, accumulating about six times more proline than the wild-type line under both saline and drought conditions. Transgenic lines 5 and 6 accumulated four times and four times more proline than the wild-type, respectively.

### 2.9. Enzymatic Activities of Ascorbate Peroxidase (APX) and Catalase (CAT)

The activities of two reactive oxygen species (ROS) scavenging enzymes, APX and CAT, were quantified in wild-type and transgenic plants under normal and stressed conditions. Under normal conditions, no significant differences were found in the activities of the enzymes in wild-type and transgenic tobacco lines (Figure 7C,D). However, under salt stress and drought conditions, the activities of both enzymes were significantly increased in transgenic lines relative to wild-type plants (Figure 7C,D).

## 3. Discussion

A lot of research has focused on understanding plants’ dehydration stress defense mechanisms for crop improvement purposes [3]. The late embryogenesis abundant (LEA) proteins were first discovered in cotton seeds [18]. The dehydrins (group 2 class of the LEA protein family) are one of the most studied gene families involved in plant dehydration stress defense [3,19]. Although there have been several studies on dehydrins in the Curcurbitaceae family, very few have focused on *Cucumis melo dehydrin* genes. For this reason, our study focuses on the cloning and functional characterization of a Y3SK2-type dehydrin, *Cucumis melo CmLEA-S* gene (Figure 1).

The protein encoded by the *CmLEA-S* gene was homologous and structurally similar to another Y3SK2 *CsLEA11* gene [12] which was found to improve abiotic stress tolerance in *E-coli* cells. Furthermore, *CmLEA-S* was upregulated in response to drought and salt stresses (Figure 1C). YnSKn dehydrins have been shown to mainly respond to salt, ABA and drought [20,21]. For example, in barley, almost all the dehydrin genes encoding for YnSK2 dehydrins were upregulated by both dehydration and ABA, but not by low temperatures. In contrast, DHN5 (K9) and DHN8 (Sk3) were upregulated by cold treatment. In vitro studies of wild-type and mutated forms of durum wheat DHN5 seemed to indicate a correlation between the number of copies of the K-segment in a dehydrin protein and the level of protection conferred by the protein under dehydration stress [22]. The K-segments or domain of dehydrins are capable of forming amphipathic α-helixes in the presence of helical inducers by binding to anionic phospholipid vesicles [23] to provide protection to cell membranes during stress [24].

Drought and salt stresses elicit a common effect of inducing physiological water deficiency in plants. Moreover, these stress factors interfere with carbon dioxide metabolism by impeding CO_2_ diffusion into the chloroplast [25]. Transgenic plants showed high germination percentages compared with wild-type plants under drought and salt stress conditions (Figure 3). Similar results were reported by [26] in transgenic *Arabidopsis* plants overexpressing the wheat *DHN-5* gene. The lengths of the primary roots of transgenic lines were also longer than that of wild-type plants under drought and salt stress conditions (Figure 4). It has been reported that transgenic tobacco lines overexpressing the *OeSRC1* dehydrin gene showed an increase in root growth relative to wild-type plants under drought and salt stresses [27]. Transgenic plants displayed fewer signs of wilting and chlorosis compared to the wild-type line (Figure 5 and Figure 6). A similar observation has been made in transgenic Chinese cabbage plants overexpressing an *LEA* gene from *Brassica napus* [28]. In conclusion, our results indicate that the *CmLEA-S* gene could confer drought and salt tolerance to transgenic tobacco plants at both germination and seedling stages.

Drought and salt stresses often cause ROS to build up in plant cells, thereby leading to MDA accumulation and lipid peroxidation [29]. As shown in Figure 7A, our results indicate that the amount of MDA in transgenic plants was significantly lower than in wild-type plants. These results are in line with the report of [30] on the protective effects of *CuCOR19*, a dehydrin, in cold stressed transgenic tobacco plants against lipid peroxidation. Based on these results, it can be suggested that *CmLEA-S* transgenic tobacco lines could perform better under stressed conditions because they experienced significantly lower levels of membrane damage compared with wild-type plants.

Plants accumulate proline in response to stresses [31]. Proline performs several functions such as the provision of osmoprotection, the stabilization of cellular structures as well as enzymes, ROS scavenging and the maintenance of redox balance in plant cells undergoing stress [32]. A significantly higher amount of free proline was observed in *CmLEA-S*-overexpressing lines compared with the wild-type line (Figure 7B). The accumulation of proline in transgenic plants conferred protection against salt and drought stresses. In support of our findings, it has been reported that maize seedlings exogenously treated with proline performed better under stress conditions [33]. It could therefore be inferred that the overexpression of the *CmLEA-S* gene enhanced the accumulation of osmoprotectants in tobacco plants.

The antioxidation system is crucial for plants’ survival against oxidative damage [34]. The accumulation of reactive oxygen species such as hydrogen peroxide has been reported to occur in plants under diverse stress conditions including drought and salinity stresses [35,36]. A high amount of hydrogen peroxide in plant tissues can pose a lot of harm to plants. APX and CAT are among the key hydrogen peroxide scavenging enzymes in plants [37]. The overexpression of *CmLEA-S* resulted in the enhanced activities of antioxidant enzymes such as APX and CAT in transgenic tobacco lines under salt and drought stresses relative to wild-type plants (Figure 7C,D). In support of our findings, it has been reported that the accumulation of APX and CAT in the tissues of drought-stressed *Panicum sumatrense* led to enhanced drought tolerance [38]. Drought- and salt-tolerant alfalfa varieties were also found to possess high antioxidant enzyme activities [39]. Two dehydrin genes, PpDHNA and PpDHNC, from moss were found to confer salinity and drought tolerance to transgenic Arabidopsis by enhancing the ROS scavenging mechanisms in transgenic plants [40].

The overexpression of *CmLEA-S* in transgenic tobacco plants resulted in enhanced drought and salt stress tolerance in tobacco seeds and seedlings, as revealed by in vitro stress analysis. It also led to enhanced drought and salt stress tolerance in tobacco plants at the early vegetative stages, as shown by in vivo experiments. The *CmLEA-S* gene, a dehydrin, could confer drought and salt tolerance to tobacco plants both in vitro and in vivo possibly through a variety of mechanisms. Dehydrins have been reported to scavenge reactive oxygen species either independently or together with ROS scavenging enzymes. Two dehydrin genes from sorghum, *SbDHN1* and *SbDHN2*, were found to actively scavenge reactive oxygen species [41]. The protective effect of the K-segment of dehydrins against oxidative damage has also been reported by [42]. Proteins tend to lose their functional properties once they become denatured. The chaperone nature of dehydrins allowed CmLEA-S proteins to possibly bind and shield ROS scavenging enzymes such as APX and CAT from aggregation and denaturation during stress thereby preserving their functions. The protective properties of the dehydrin protein may have also extended to cell membranes, where they may have bound to the lipid bilayer to prevent MDA accumulation and lipid peroxidation, thus preserving the integrity of cells and membranes under stress.

## 4. Materials and Methods

### 4.1. Sequence Alignment and Phylogenetic Analysis of CmLEA-S

The sequence information of a *Cucumis melo* Y3SK2-type LEA gene, designated as *CmLEA-S*, was retrieved from the NCBI database (Gen-Bank Accession Number: XP_008460975). Amino acid sequences of other Y3SK2-type LEA proteins of *Cucumis sativus* (*CsLEA11*, XP_004150075), *Brassica napus* (XP_013660957), *Nicotiana tabacum* (NtLEA-S from N. sylvestris, XM_016583816 and NtLEA-T from N. tomentosiformis, XM_016625068), *Manihot esculenta* (XP_021628236), *Gossypium raimondii* (KJB77750), *Ipomoea nil* (XP_019165508), *Vitis vinifera* (RVW48259), *Helianthus annua* (X92647) and *Arabidopsis thaliana* (NP_179744) were also collected from the GenBank and aligned with the CmLEA-S protein using the Clustal W software [43]. A phylogenetic tree was constructed with the MEGA 5.0 software by the neighbor joining method with 1000 bootstrap replicates [44].

### 4.2. Expression Analysis of CmLEA-S in Melon Plants

Three replicates of melon plants were grown under normal conditions for 2 weeks. Afterwards, one replicate of plants was maintained under normal watering conditions, and the other two sets were subjected to drought and salt stress treatments for seven days. RNAs were extracted from leaf, shoot and root samples of unstressed, salt-stressed, and drought-stressed melon plants for cDNA synthesis. cDNAs were subsequently subjected to qPCR analysis on a Step One Plus Real-Time PCR System machine (Applied Biosystems) under the following conditions: initial denaturation at 98 °C for 2 min, denaturation at 98 °C for 10 s, annealing at 60 °C for 10 s and extension 68 °C at 30 s for 40 cycles. Gene expression data (Ct values) were evaluated using the 2^−∆∆CT^.

The *CmEF1a* gene was used as the internal control gene.

### 4.3. Cloning of CmLEA-S and Agrobacterium-Mediated Transformation of Tobacco Plants

Total RNA was isolated from melon leaves with the aid of the RNA iso plus kit (TaKaRa Bio Inc., Japan). *CmLEA-S* cDNA (570 bp) was amplified with gene specific primers (CmLEA5P, CmLEA3P, Supplementary Materials Table S1), transferred into the PCR8 entry vector and then cloned in between the attR1 and attR2 sites of the pGWB2 binary vector [45]. The recombinant pGWB2 35SP: *CmLEA-S* contained the 35S promoter to drive the expression of the *CmLEA-S* gene, with the hygromycin phosphotransferase (*hpt*) and neomycin phosphotransferase (*nptII*) genes as selectable marker genes. The recombinant vector was transformed into tobacco leaf discs (*Nicotiana tabacum* ‘Petit Havana’) using the *Agrobacterium tumefaciens* strain GV3101.

Putative transgenic shoots regenerated on hygromycin media were analyzed using Southern blot analysis. Briefly, genomic DNAs (10 µg) were isolated from transgenic shoots according to the cetyltrimethyl ammonium bromide (CTAB) method of [46] and digested overnight with *Hin*dIII (TaKaRa) at 37 °C. The digested DNA fragments were separated on 0.8% agarose gel and transferred to a nylon membrane. Hygromycin phosphotransferase (*hpt*) probe (about 600 bp) was labeled using PCR DIG Probe Synthesis Kit (Roche). Probe hybridization, stringency washes and chemiluminescence detection with CDP-Star were performed according to the manufacturer’s instructions [47].

Putative transgenic shoots were analyzed by reverse transcription polymerase chain reaction (RT-PCR) using primers for the *CmLEA-S* gene (Table S1) under the following PCR conditions: initial denaturation at 94 °C for 2 min, denaturation at 94 °C, for 10 s, annealing at 55 °C for 10 s and extension at 68 °C for 15 s. Amplifications were carried out for 30 cycles and the elongation factor 1a (*EF-1a*) gene was used as an internal control. Afterwards, selected transgenic shoots were rooted, acclimatized and transferred to the green house to obtain T_2_ seeds. Plants were covered at the flowering stage with paper bags to prevent cross-pollination.

### 4.4. Stress Tolerance Analysis at the Germination Stage

To compare the responses of wild-type and transgenic seeds under normal and drought conditions, 60–70 wild-type and T_2_ seeds of transgenic plants were grown on filter papers soaked in water and 10% PEG. PEG was applied to osmotically induce drought stress in seeds. Similarly, to evaluate the performance of wild-type and transgenic lines under normal and salt stress conditions, seeds of both wild-type and transgenic plants were grown on normal MS media and MS media supplemented with 150 mM NaCl. Each experiment was repeated thrice and the seed germination rate in each treatment computed 20 days after sowing.

### 4.5. Stress Tolerance Assays at the Seedling Stage

For drought stress assays at the seedling stage in vitro, 2 week-old seedlings were transferred to normal MS media and MS media supplemented with 300/400 mM mannitol (mannitol was added to the medium to osmotically induce water deficit stress in the growth environment of tobacco seedlings) and allowed to grow until the fourth week. Subsequently, root length and fresh weight measurements were taken for ten individual plants in each treatment. In the same way, the in vitro salt stress assay was performed by growing 2 week-old young seedlings on normal MS and MS media supplemented with 250/300 mM NaCl, which were allowed to grow for an additional 2 weeks, and root length and fresh weight measurements were taken for ten plants in each treatment. Leaf samples were later taken from plants in each treatment and stored at −80 °C for RNA extraction.

### 4.6. Stress Tolerance Assays on Soil

Two week-old tissue cultured tobacco seedlings were acclimatized in a growth chamber on sandy loam soils at a temperature of 25 °C under a 16/8 h photoperiod. At 3 weeks, plants were moved to the greenhouse. Plants were watered equally every 3 days until stress application. Drought stress analyses were performed on 4 week-old tobacco plants. For drought stress treatment, wild-type and transgenic plants (20 plants per line) were treated with 20% PEG for 10 days. Salt stress was induced in 5 week-old tobacco plants (20 plants per line) by irrigating plants with 300 mM NaCl solution for 21 days. Parameters such as percentage of wilted and chlorotic leaves were determined respectively in drought- and salt-stressed plants at the end of the experiments.

### 4.7. Analysis of Water Loss from Leaves

The rate of water loss was determined in uniform and healthy leaves (the second upper leaf) of wild-type and transgenic plants growing under normal conditions according to the method described by [48,49]. Plant leaves were placed on filter papers at room temperature and left to dehydrate for 3 h. Measurements for the fresh weight (LWt) of leaves were taken at every 60 min interval in three replicates. The relative water loss (RWL) in leaves after exposure was determined as follows: RWL = (FW-LWt)/FW × 100, where LWt is the weight of the leaf subjected to desiccation treatment for t hours, and FW is the weight of leaves before desiccation.

### 4.8. Measurement of Malondialdehyde (MDA) and Proline Contents

MDA and proline contents were determined in leaves of the same size sampled from unstressed and stressed wild-type and transgenic plants. The MDA content of leaves was determined according to the method described previously by [50]. Briefly, 0.5 g of fresh tobacco leaves were ground in liquid nitrogen and homogenized with 20mL of 10% trichloroacetic acid (TCA). The homogenate was centrifuged at 5000× *g* for 10 min and the supernatant collected. Then, 1 mL of the supernatant was taken and added to 3 mL of 0.5% thiobarbituric acid (TBA) (0.5% TBA was prepared by dissolving TBA in 10% TCA) and mixed. The reaction mixture was then incubated in water bath at 95 °C for 20 min. At the end of the heat treatment, the mixture was cooled immediately on ice and centrifuged at 10,000× *g* for 10 min to cause debris in the mixture to settle. Spectrophotometric measurements were taken at 532 nm and subtracted from the non-specific absorbance at 600 nm. TBARS were determined with an extinction coefficient of 155 m/M/cm. The thiobarbituric acid reactive substance (TBARS) content was determined according to the formula:

(1) $$\begin{array}{l} \mathrm{TBARS}\;\mathrm{content}\;\left[\mathrm{nmol}\;\mathrm{MDA}\frac{\mathrm{equivalents}}{g}\mathrm{fresh}\;\mathrm{weight}\left(\mathrm{FW}\right)\right]\\ \;\;\;\;\;\;\;=\;\left(\mathrm{OD}532\;-\;\mathrm{OD}600\right)\times Vt\times Vr\times 1000/\left(Vs\times m\times 155\right) \end{array}$$

where *Vt* is the total volume of the extract solution, *Vr* is the total volume of the reaction mixture solution, *Vs* is the volume of the extract solution contained in the reaction mixture solution, and *m* was the mass of samples.

Proline concentration of leaves was determined in accordance with the method described by [51]. Briefly, 0.5 g of fresh tobacco leaf samples were homogenized in 3% sulfosalicylic acid. The reaction mixtures were then centrifuged, and the supernatants collected. Then, 2 mL of the supernatant was added to 2 mL each of glacial acetic acid and ninhydrin reagent and vortexed. Subsequently, the reactions were incubated at 100 °C for an hour, after which the reaction was terminated on ice. Four milliliters of toluene was added and the supernatants collected for spectrophotometric reading. Spectrophotometric readings were taken for three biological replicates. The relative amount of proline was determined on a standard curve.

### 4.9. Determination of APX and CAT Activities.

Two milliliters of ice-cold 50 mM phosphate buffer (pH 7.8) containing 1 mM EDTA was used to homogenize 0.20 g of frozen leaves in a mortar. The homogenate was centrifuged at 15,000× *g* for 15 min at 4 °C and the supernatant used to assay the activities of APX (EC1.11.16) and CAT (EC1.11.1.6).

APX activity was assayed following the method described by [52]. Briefly, a 3 mL reaction mixture containing 50 mM phosphate buffer (pH 7.8), 0.1 mM EDTA, 0.5 mM ascorbate, 0.1 mM hydrogen peroxide (H_2_O_2_) and 0.2 mL enzyme extract was used. The reaction was initiated with H_2_O_2_ and ascorbate oxidation measured at 30 s intervals at 290 nm for 3 min. APX activity was quantified using the molar extinction coefficient for ascorbate 2.8 mM^−1^ cm^−1^.

Catalase activity was determined in accordance with the method described by [53]. Briefly, 3 mL of reaction solution consisting of 100 mM phosphate buffer (pH7.0), 0.1 mM EDTA, 0.1% H_2_O_2_ and 0.2 mL enzyme extract was added to initiate the reaction and the rate of decrease in hydrogen peroxide measured at 240 nm and quantified at an extinction co-efficient of 39.4 mM^−1^ cm^−1^.

#### Statistical Analysis

Data analysis was performed with the SPSS software (SPSS Inc., Chicago, IL, USA). Duncan’s test was used to separate differences in means between treatments at a probability level of 0.05. Graphs were generated with Microsoft Excel. All experiments were performed in triplicate.

## 5. Conclusions

The molecular breeding of crops with elite genes is one major way by which the challenge of food security could be addressed. In this work, we have presented both in vitro and in vivo evidence to show that the *CmLEA-S* gene could confer drought and salt stress tolerance to transgenic tobacco plants at the germination, seedling and vegetative stages of plants life cycle by enhancing osmoprotectant accumulation, antioxidation enzyme activity and inhibiting membrane damage. Considering the level of protection, the gene could confer to transgenic tobacco plants, and *CmLEA-S* could be a valuable resource for future crop improvement programs.

## Supplementary Materials

The following are available online at https://www.mdpi.com/2223-7747/9/12/1749/s1, Table S1: List of primers.
