# Supplementary material for: Over-Expression of a Melon Y3SK2-Type LEA Gene Confers Drought and Salt Tolerance in Transgenic Tobacco Plants
Source: Plants (Basel). 2020 Dec 10;9(12):1749. doi: 10.3390/plants9121749 (PMC7763651; doi:10.3390/plants9121749)
Supplement: Supplementary file 1 [file plants-09-01749-s001.pdf]

Supplementary Table 1 List of primers used in this study

Primer names	Sequences (5' - 3')
CmLEA5P	TACAAAGCATATAATCTACAATGG
CmLEA3P	ACATATATACTAAATAAGTTTCCA
CmLEART5P	AAGAGAAGTTAACCGGAAGTGGGA
CmLEART3P	TTTTCCATAACCCCCTTCTTCCCA
CmEF1a5P	GTCAAGCAGATGATCTGCTGTTGT
CmEF1a3P	TCGGGGTTGTAACCAACCTTCTTC
NtEF1a5P	ACTGTGCTGTCCTGATTATTGACT
NtEF1a3P	GGACCAAAAGTAACAACCATACCA
HPT5P	CAGCTTCGATGTAGGAGGGCGTGG
HPT3P	AATCCCCGAACATCGCCTCGCTCC
